# Regulation of the SIAH2-HIF-1 Axis by Protein Kinases and Its Implication in Cancer Therapy

**DOI:** 10.3389/fcell.2021.646687

**Published:** 2021-03-25

**Authors:** Dazhong Xu, Cen Li

**Affiliations:** Department of Pathology, Microbiology and Immunology, School of Medicine, New York Medical College, Valhalla, NY, United States

**Keywords:** protein kinases, SIAH2, hypoxia, HIF-1, cancer, therapy

## Abstract

The cellular response to hypoxia is a key biological process that facilitates adaptation of cells to oxygen deprivation (hypoxia). This process is critical for cancer cells to adapt to the hypoxic tumor microenvironment resulting from rapid tumor growth. Hypoxia-inducible factor 1 (HIF-1) is a transcription factor and a master regulator of the cellular response to hypoxia. The activity of HIF-1 is dictated primarily by its alpha subunit (HIF-1α), whose level and/or activity are largely regulated by an oxygen-dependent and ubiquitin/proteasome-mediated process. Prolyl hydroxylases (PHDs) and the E3 ubiquitin ligase Von Hippel-Lindau factor (VHL) catalyze hydroxylation and subsequent ubiquitin-dependent degradation of HIF-1α by the proteasome. Seven *in Absentia* Homolog 2 (SIAH2), a RING finger-containing E3 ubiquitin ligase, stabilizes HIF-1α by targeting PHDs for ubiquitin-mediated degradation by the proteasome. This SIAH2-HIF-1 signaling axis is important for maintaining the level of HIF-1α under both normoxic and hypoxic conditions. A number of protein kinases have been shown to phosphorylate SIAH2, thereby regulating its stability, activity, or substrate binding. In this review, we will discuss the regulation of the SIAH2-HIF-1 axis via phosphorylation of SIAH2 by these kinases and the potential implication of this regulation in cancer biology and cancer therapy.

## Introduction

Cellular response to low oxygen tension (hypoxia) is one of the most fundamental biological processes that determine the survival of cells under the hypoxic condition (Bruick, 2003; Kaelin and Ratcliffe, 2008). Although the hypoxic response is part of the normal cellular reaction to hypoxic stress under physiological settings, mounting evidence also supports its pivotal role in tumor angiogenesis to supply the poorly vascularized tumor mass and in reprogramming cancer cells to adapt to the hypoxic tumor microenvironment. These activities facilitate cancer progression by regulating the survival, metabolism, immune evasion, and metastasis of cancer cells (Carmeliet et al., 1998; Semenza, 2000, 2003; Harris, 2002; Liao and Johnson, 2007; Eales et al., 2016; Samanta and Semenza, 2018). The hypoxic response employs a complex intracellular signaling network with hypoxia-inducible factors (HIFs) as its central elements (Bruick, 2003; Kaelin and Ratcliffe, 2008; Semenza, 2012a). HIFs are transcription factors that are induced and activated in response to low oxygen tension (Semenza, 2012a). Induction and activation of these factors by hypoxia lead to the expression of a spectrum of genes that contribute to the overall hypoxic response (Bruick, 2003; Kaelin and Ratcliffe, 2008; Semenza, 2012a). Rapid progress in research has revealed increasingly complex regulations of this signaling pathway since its discovery 30 years ago (Semenza et al., 1991).

The HIF transcription factors consist of two subunits: the alpha subunit (HIF-α) and the shared beta subunit (HIF-β or ARNT) (Semenza, 1999; Bruick, 2003; Kaelin and Ratcliffe, 2008). There are three mammalian alpha subunits, HIF-1α, HIF-2α, and HIF-3α. HIF-1α and HIF-2α are structurally similar with somewhat redundant functional profiles while HIF-3α are a number of splice variants, some of which function as dominant-negative inhibitors of HIF-1α or HIF-2α (Majmundar et al., 2010). The level and activity of HIF transcription factors are primarily controlled by their alpha subunits, whose expression and activity are regulated mainly at the post-translational level in response to hypoxia and some other conditions (Bruick, 2003; Kaelin and Ratcliffe, 2008; Semenza, 2012a). In contrast, HIF-β is constitutively expressed (Semenza, 1999; Bruick, 2003; Kaelin and Ratcliffe, 2008). Under normoxia, HIF-1α and HIF-2α are subjected to oxygen-dependent hydroxylation mediated by prolyl hydroxylases (PHDs). Hydroxylation promotes their ubiquitination, which is catalyzed by the E3 ubiquitin ligase Von Hippel-Lindau Factor (VHL), and subsequent degradation by the ubiquitin proteasome system (UPS) (Bruick, 2003; Kaelin and Ratcliffe, 2008; Semenza, 2012a). Hypoxia inhibits the hydroxylation because of reduced activity of PHDs and leads to stabilization of HIF-1α and HIF-2α. HIF-1 is the most studied among HIFs due to its wide tissue distribution and its involvement in more pathophysiological conditions (Weidemann and Johnson, 2008). In addition to VHL-dependent degradation, HIF-1α is also regulated by other mechanisms, including phosphorylation and SUMOylation (Kietzmann et al., 2016; Filippopoulou et al., 2020).

Past work has revealed the regulation of the HIF-1 pathway by Seven *in Absentia* Homolog 2 (SIAH2), a ubiquitin E3 ligase. SIAH2 indirectly regulates the level of HIF-1α by promoting degradation of PHDs by UPS (Nakayama et al., 2004, 2009). SIAH2, in turn, is positively regulated by hypoxia-dependent transcriptional and posttranscriptional mechanisms (Nakayama et al., 2009). SIAH2 is also directly regulated by several protein kinases through phosphorylation (Khurana et al., 2006; Calzado et al., 2009; Perez et al., 2012; Sarkar et al., 2012; Garcia-Limones et al., 2016; Li et al., 2017). In this review, we will discuss the regulation of the SIAH2-HIF-1 pathway by protein kinases and its potential implications in cancer biology and therapy.

## The SIAH2-HIF-1 Axis

HIF-1 is a central regulator of the hypoxic response, with strong implications in cancer (Semenza, 1999; Bruick, 2003; Kaelin and Ratcliffe, 2008; Weidemann and Johnson, 2008). As a transcription factor, HIF-1 propels expression of a group of genes whose promoters contain the hypoxia response element. Examples of these genes are insulin-like growth factor 2 (IGF2), BCL2/adenovirus E1B 19 kDa protein-interacting protein 3 (BNIP3), erythropoietin (EPO), mesenchymal-epithelial transition factor (C-Met), vascular endothelial growth factor (VEGF), and glucose transporter 1 (GLUT1) (Semenza, 1999; Bruick, 2003; Kaelin and Ratcliffe, 2008; Weidemann and Johnson, 2008). Expression of these genes affects a broad array of cellular activities, including proliferation, survival, neoangiogenesis, migration, and metabolism (Semenza, 1999, 2012a,b, 2016, 2017; Bruick, 2003; Kaelin and Ratcliffe, 2008). These cellular activities have profound pathophysiological consequences.

In normoxia, HIF-1α is constantly hydroxylated by PHDs (PHD1-3) at proline 402 and 564 in an oxygen-dependent fashion. Hydroxylated HIF-1α is subjected to degradation by UPS with VHL as its E3 ubiquitin ligase (Semenza, 1999; Bruick, 2003; Kaelin and Ratcliffe, 2008). In hypoxia, low oxygen tension inhibits HIF-1α hydroxylation by PHDs, thereby preventing VHL-mediated degradation of HIF-1α by UPS. Stabilization of HIF-1α leads to higher levels of the HIF-1α/HIF-β dimer and HIF-1-mediated transcription in the cell (Semenza, 1999; Bruick, 2003; Kaelin and Ratcliffe, 2008). Oxygen-dependent hydroxylation of HIF-1α also takes place at the residue asparagine 803, which is mediated by the asparaginyl hydroxylase factor inhibiting HIF (FIH) (Lando et al., 2002). Rather than affecting the stability of HIF-1α, hydroxylation of this residue inhibits the transcriptional activity of HIF-1 by disrupting the recruitment of transcriptional cofactors and histone acetyltransferase p300/CBP to its C-terminal transactivation domain (C-TAD) (Lando et al., 2002). Other posttranslational modifications, such as phosphorylation and SUMOylation, also regulate the stability and/or nuclear translocation of HIF-1α (Richard et al., 1999; Sodhi et al., 2000; Mylonis et al., 2006, 2008; Carbia-Nagashima et al., 2007; Cheng et al., 2007; Flugel et al., 2007; Yang et al., 2008; Xu et al., 2010, 2017; Warfel et al., 2013). The kinases that directly phosphorylate HIF-1α include ERK1/2, p38, GSK3β, PLK3, and CDK1 (Richard et al., 1999; Sodhi et al., 2000; Mylonis et al., 2006; Flugel et al., 2007; Xu et al., 2010, 2017; Warfel et al., 2013).

**FIGURE 1:**
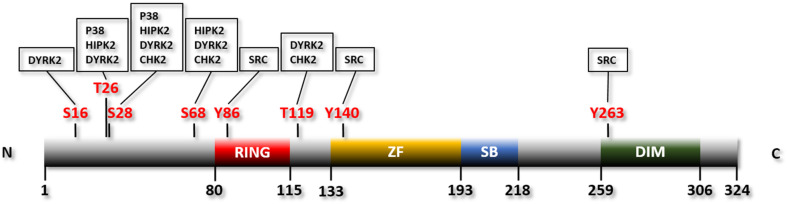
Linear structure of human SIAH2 with the phosphorylation residues and the protein kinases indicated. RING, RING finger domain; ZF, zinc finger domain; SB, substrate binding domain; DIM, dimerization domain.

SIAH2 is a member of a small and evolutionarily conserved group of RING finger ubiquitin E3 ligases (Metzger et al., 2014; Pepper et al., 2017). The prototype of them is Seven *in Absentia* (SINA) in *Drosophila.* This protein is required for the formation of the R7 photoreceptor (Pepper et al., 2017). There are three vertebrate orthologs of SINA: SIAH1, SIAH2, and SIAH3. SINA or its ortholog SIAH1 exists in most metazoans whereas SIAH2 and SIAH3 are restricted to vertebrates (Pepper et al., 2017). SIAH3 lacks a functional RING finger and is therefore considered an inactive E3 ligase (Pepper et al., 2017). Mouse SIAH1 has two isoforms encoded by two separate genes, SIAH1A and SIAH1B (Della et al., 1993). While SIAH1A and SIAH1B share 98% homology, SIAH1 and SIAH2 are much less identical (77% homology) (Della et al., 1993). Deletion of *Siah1a* in mice leads to severe growth retardation and frequent early lethality (Dickins et al., 2002). In contrast, *Siah2* knockout mice are largely normal with only mild phenotypic changes, e.g., increased number of hematopoietic progenitor cells (Frew et al., 2003). Double deletion of *Siah1* and *Siah2*, on the other hand, results in embryonic lethality (Nakayama et al., 2009). Human SIAH2 contains 324 amino acids, with a RING domain located at the N-terminal region for recruiting the E2 ubiquitin conjugating enzyme, a zinc finger domain and a substrate binding domain located C-terminal to the RING domain, and a dimerization domain located at the C-terminus of the protein (Reed and Ely, 2002; Pepper et al., 2017; Figure 1).

SIAH2 can function as an E3 ubiquitin ligase either alone or in complex with other proteins (e.g., Siah-interacting protein and phyllopod) (Matsuzawa and Reed, 2001; Li et al., 2002). It targets multiple proteins for degradation by UPS, thereby regulating a number of cellular pathways. For instance, it activates the Ras signaling pathway by destabilizing Sprouty 2, promotes cell cycle progression by destabilizing p27, suppresses the oxidative stress response by destabilizing NRF1 and NRF2, regulates the tumor necrosis factor alpha pathway by destabilizing TRAF2, inhibits the DNA damage response by destabilizing CHK2, reduces P53 acetylation and activity by mediating ubiquitination and degradation of its acetyltransferase, and activates the HIF-1 pathway by destabilizing PHDs (Habelhah et al., 2002; Nadeau et al., 2007; Grishina et al., 2012; Baba et al., 2013; Qi et al., 2013; Garcia-Limones et al., 2016; Ma et al., 2019).

The regulation of the HIF-1 pathway and hypoxic response by mediating proteasomal degradation of PHDs is one of the most prominent and well-established functions of SIAH2 (Nakayama and Ronai, 2004; Nakayama et al., 2004, 2009; Qi et al., 2013). The interaction between SIAH2 and PHDs was discovered by an unbiased mass spectrometric study aiming to identify SIAH2-associated proteins (Nakayama et al., 2004). The study further demonstrated that SIAH2 mediates ubiquitination and proteasomal degradation of PHD1 and PHD3 (PHD3 in particular), thereby stabilizing HIF-1α and promoting HIF-1-mediated transcription. Hypoxia, in turn, induces mRNA expression of SIAH2 likely via PI3K/AKT-dependent downstream transcriptional events, as inhibition of PI3K attenuates the induction (Nakayama and Ronai, 2004; Nakayama et al., 2004, 2009; Qi et al., 2013). This feed-forward mechanism contributes to the rapid increase of the HIF-1α level in response to hypoxia (Nakayama and Ronai, 2004; Nakayama et al., 2004, 2009; Qi et al., 2013). Surprisingly, no evidence indicates that HIF-1-mediated transcription is responsible for the induction of SIAH2 by hypoxia. Also interesting is that although SIAH2 also interacts with PHD2 (albeit much weaker than PHD1 and PHD3), it does not affect its stability (Nakayama et al., 2004). The mechanism underlying the resistance of PHD2 to SIAH2-mediated degradation remains to be determined. Of note, SIAH2 acts alone as the E3 ligase of PHDs rather than as a member of the E3 ligase complex (Nakayama et al., 2004). Moreover, SIAH2 promotes its own degradation via auto-ubiquitination (Qi et al., 2008).

## Regulation of SIAH2 by Protein Kinases

A number of protein kinases have been shown to phosphorylate SIAH2. These include serine/threonine kinases p38, HIPK2, DYPK2, CHK2, and PLK3, as well as the tyrosine kinase SRC. A recent report suggests that SIAH2 may also be phosphorylated by MRCKβ, although more direct evidence is needed to confirm this (Dixit et al., 2021). Phosphorylation of SIAH2 affects its stability, enzymatic activity as an E3 ubiquitin ligase, or subcellular localization. Regulation of SIAH2 by all these kinases but SRC has been linked to HIF-1 signaling and the hypoxic response.

### Regulation of SIAH2 by p38

The p38 kinase is a key MAP kinase in one of the four MAP kinase pathways (i.e., ERK1/2, JNK, p38, and ERK5 pathways) (Morrison, 2012). The p38 pathway is activated in response to a wide variety of cellular signals, including cellular stress, growth factors, and inflammatory cytokines (Kyriakis and Avruch, 2012). The involvement of this signaling pathway in cancer, albeit complex, has been well established (Wagner and Nebreda, 2009; Kyriakis and Avruch, 2012; Igea and Nebreda, 2015). Available evidence indicates that p38 can be a tumor suppressor or a tumor promoter depending on the tissue types and participate in multiple stages of tumorigenesis, from tumor initiation to metastasis (Igea and Nebreda, 2015).

The p38 MAPK pathway is activated by mitochondria-dependent production of reactive oxygen species (ROS) in hypoxia (Kulisz et al., 2002; Emerling et al., 2005). p38, in turn, phosphorylates and activates SIAH2 (Khurana et al., 2006). Consistently, pharmacological inhibition of p38 reduces phosphorylation of SIAH2 (Khurana et al., 2006). p38 phosphorylates mouse Siah2 at residues threonine 24 and serine 29 (Khurana et al., 2006; Figure 1 and Table 1). Of note, threonine 24 and serine 29 of mouse Siah2 are equivalent to threonine 26 and serine 28 of human SIAH2, respectively (Hu et al., 1997). Phosphorylation of SIAH2 by p38 increases its E3 ligase activity toward PHD3 without affecting its self-ubiquitination (Khurana et al., 2006). Interestingly, while both of these residues are phosphorylated by p38, only the phosphorylation of serine 29 enhances the activity of SIAH2 toward PHD3. Furthermore, the phospho-defective mutants of SIAH2, with the two residues mutated to alanine, are unable to bind to PHD3, suggesting that phosphorylation of these residues is critical for the interaction between the two proteins (Khurana et al., 2006). Phosphorylation of these two residues may also affect the subcellular localization of SIAH2. While the wild-type SIAH2 can be found in both the nucleus and cytoplasm, SIAH2 with both threonine 24 and serine 29 mutated to aspartate, which mimics the phosphorylated form of SIAH2, are mainly located in the perinuclear region (Khurana et al., 2006). In contrast, the phospho-defective mutant is located mainly in the nucleus and unable to bind to PHD3 (Khurana et al., 2006). The exclusion of phosphorylated SIAH2 from the nucleus is apparently due to active export (Khurana et al., 2006). As SIAH2-mediated degradation of PHD3 mainly takes place in the nucleus, the increased activity and exclusion of SIAH2 from the nucleus seems counterproductive. It was proposed that phosphorylated SIAH2 is able to mediate PHD3 degradation before exported from the nucleus by the constitutive nuclear export mechanism (Khurana et al., 2006). Phosphorylation of SIAH2 by p38 is apparently part of the mechanism that regulates HIF-1 in response to hypoxia. Thus, p38 is activated by hypoxia-induced generation of ROS and subsequently phosphorylates and activates SIAH2. SIAH2, in turn, promotes degradation of PHD3, thereby stabilizing HIF-1α (Kulisz et al., 2002; Emerling et al., 2005; Khurana et al., 2006; Nakayama et al., 2009).

**TABLE 1:** Phosphorylation sites of SIAH2 by protein kinases.

Kinase	Phosphorylated residue	References
P38	T26 and S28	Khurana et al., 2006
HIPK2	T26, S28, and S68	Calzado et al., 2009
DYRK2	S16, T26, S28, S68, and T119	Perez et al., 2012
CHK2	S28, S68, and T119	Garcia-Limones et al., 2016
PLK3	TBD	Li et al., 2017
SRC	Y86, Y140, and Y263	Sarkar et al., 2012

### Regulation of SIAH2 by HIPK2

Homeodomain-interacting protein kinase 2 (HIPK2) is a serine/threonine kinase of the expanded dual-specificity tyrosine-regulated kinase (DYRK) family, which consists of four members: HIPK1-4 (Blaquiere and Verheyen, 2017; Feng et al., 2017). HIPK2 is predominantly a nuclear kinase localized at nuclear bodies and partly overlaps with polycomb protein bodies (Roscic et al., 2006; Sombroek and Hofmann, 2009). It has significant functions in cell proliferation, apoptosis, and the DNA damage response (Sombroek and Hofmann, 2009; Feng et al., 2017). HIPK2 phosphorylates p53 at serine 46 thereby activating the transcription of p53 target genes, cell cycle checkpoints, and apoptosis (Sombroek and Hofmann, 2009). It also plays a role in TGF-β-mediate apoptosis by activating the JNK pathway (Hofmann et al., 2003). HIPK2 suppresses β-catenin-mediated transcription by promoting degradation of CtBP, a component of the transcriptional machinery, thereby negatively regulating the Wnt/BMP signaling pathway (Zhang et al., 2003; Wei et al., 2007). HIPK2 may also act as a suppressor of transcription to inhibit transcription of HIF-1α, thereby inhibiting VEGF expression and angiogenesis (Choi et al., 1999; Nardinocchi et al.,2009a,b). These functions suggest a significant role of HIPK2 in cancer (Sombroek and Hofmann, 2009). Indeed, a small subset of acute myeloid leukemia and myelodysplastic syndrome patients contain mutations of HIPK2 (Li et al., 2007). HIPK2 knockout leads to carcinoma *in situ* or invasive squamous cell carcinoma in a two-stage skin carcinogenesis model (Wei et al., 2007).

HIPK2 is able to interact with both SIAH1 and SIAH2 (Winter et al., 2008; Calzado et al., 2009). Although both SIAH1 and SIAH2 promote HIPK2 ubiquitination and degradation by the proteasome, SIAH2 has a stronger capacity of doing so (Winter et al., 2008; Calzado et al., 2009). HIPK2 phosphorylates SIAH2 at its threonine 26, serine 28, and serine 68 (Calzado et al., 2009; Figure 1 and Table 1). Phosphorylation of these three residues destabilizes SIAH2 without affecting its E3 ligase activity (Calzado et al., 2009). The phosphorylation also greatly inhibits the binding between SIAH2 and HIPK2, following the so-called “kiss and run” mechanism (Calzado et al., 2009). The binding of SIAH2, but not SIAH1, to HIPK2 markedly increases under hypoxia, which leads to a significantly higher level of ubiquitination and destruction of HIPK2. This observation indicates that SIAH2 but not SIAH1 is more relevant to the hypoxic response in this setting (Calzado et al., 2009). Mutation of the three HIPK2 phosphorylation sites of SIAH2 abolishes the hypoxia-induced interaction of SIAH2 and HIPK2 (Calzado et al., 2009). These findings demonstrate a complex and interactive relationship of these two proteins, which coordinately regulates the cellular response to hypoxia.

### Regulation of SIAH2 by DYRK2

Dual-specificity tyrosine-regulated kinase 2 (DYRK2) is a member of the DYRK family protein kinases containing 4 members in mammals: DYRK1A, DYRK1B, DYRK2, DYRK3, and DYRK4 (Becker and Joost, 1999; Aranda et al., 2011; Soppa and Becker, 2015; Correa-Saez et al., 2020). DYRK2 functions in the DNA damage response by phosphorylating p53 at serine 46, thereby promoting cell apoptosis in response to genotoxic stress (Taira et al., 2007). DYRK2 also phosphorylates c-Jun and Myc, thereby promoting their degradation by UPS and inhibiting cell cycle progression and cell proliferation (Taira et al., 2012). Other functions of DYRK2 include acting as a scaffold for the EDVP E3 ubiquitin ligase complex to control cell cycle progression through the G2/M phase and regulating the Hedgehog signaling pathway by promoting the proteasomal degradation of the transcription factor GLI2 through direct phosphorylation (Maddika and Chen, 2009; Singh and Lauth, 2017). DYRK2 also phosphorylates NFAT, thereby priming its phosphorylation by GSK3β and CK1 (Gwack et al., 2006). Although the molecular function of DYRK2 suggests its tumor suppressive role, it may act as a tumor promoter in “proteasome-addicted” tumors by increasing proteasome-mediated protein degradation through phosphorylation of Rpt3, the 19S subunit of the proteasome (Guo et al., 2016; Banerjee et al., 2019).

DYRK2 phosphorylates SIAH2 at least 5 residues: serine 16, threonine 26, serine 28, serine 68, and threonine 119 (Perez et al., 2012; Figure 1 and Table 1). Phosphorylation of SIAH2 by DYRK2 does not affect its E3 ligase activity but inhibits its nuclear localization. Phosphorylated SIAH2 exhibits increased ability to mediate PHD3 degradation, which increases the level of HIF-1α. As a result, expression of the downstream targets of HIF-1 and angiogenesis, measured by tube formation by human umbilical vascular endothelial cells (HUVEC), also increase (Perez et al., 2012). Conversely, phosphorylation by DYRK2 destabilizes SIAH2. This is likely mediated by another E3 ubiquitin ligase, as the auto-ubiquitination activity is not affected by mutation of the phosphorylation sites (Perez et al., 2012). SIAH2, in turn, promotes ubiquitination and degradation of DYRK2 by the proteasome independent of the kinase activity of DYRK2 (Perez et al., 2012). This mechanism contributes to the decrease in DYRK2 expression in response to hypoxia and the reduced p53 phosphorylation at serine 46 in response to doxorubicin under hypoxia (Perez et al., 2012). The two regions through which SIAH2 interacts with DYRK2 were mapped using a peptide array analysis: an N-terminal domain (AA1-28) and a region partially overlapping the zinc finger domain (Perez et al., 2012). These results indicate a mutual regulatory mechanism, in which SIAH2 inhibits DYRK2 by promoting its degradation while DYRK2 reduces the SIAH2 protein level by triggering its degradation. In addition, phosphorylation by DYRK2 promotes nuclear exclusion of SIAH2 and enhances the E3 ligase activity of SIAH2 toward PHD3.

### Regulation of SIAH2 by CHK2

CHK2 is a cell cycle checkpoint kinase with important functions in the DNA damage response. It is activated in response to DNA damage via phosphorylation by ATM and subsequently phosphorylates and activates its downstream targets, such as p53, Cdc25A, and BRCA1 (Bartek and Lukas, 2003). CHK2 contributes to the initiation of DNA repair and apoptosis following DNA damage and thus plays an important role in cancer as a tumor suppressor (Bartek and Lukas, 2003). Deletion and missense mutation variants (e.g., 1100delC and I157T) of CHK2 are associated with Li-Fraumeni syndrome, a familial cancer syndrome characterized by multiple tumors at young age, as well as breast and colon cancer (Bartek and Lukas, 2003). Of note, CHK2 is phosphorylated and activated by hypoxia and participates in hypoxia-induced DNA damage response (Gibson et al., 2005).

CHK2 phosphorylates SIAH2 at three residues: threonine 26, serine 28, and threonine 119. The phosphorylation inhibits the activity of SIAH2 toward known SIAH2 substrates including PHD3 (Garcia-Limones et al., 2016). Thus, phosphorylation of SIAH2 by CHK2 stabilizes PHD3 thereby suppressing the activity of the HIF-1-dependent erythropoietin promoter, presumably by increasing the degradation of HIF-1α (Garcia-Limones et al., 2016). SIAH2, in turn, mediates ubiquitination and degradation of CHK2 via UPS. Interestingly, phosphorylation of SIAH2 by CHK2 does not affect its stability or its activity toward CHK2 (Garcia-Limones et al., 2016).

### Regulation of SIAH2 by PLK3

Polo-like kinase 3 (PLK3) belongs to a small family of evolutionarily conserved serine/threonine protein kinases. Five mammalian members of the Polo-like kinase family (PLK1-PLK5) have been identified, all of which contain a highly conserved kinase domain (KD) at the N-terminus and a polo-box domain (PBD) at the C-terminus. While the kinase domain confers catalytic activity, the polo-box domain mediates subcellular localization and the substrate binding of PLKs (Archambault and Glover, 2009; de Carcer et al., 2011b; Xu et al., 2012; Zitouni et al., 2014). PLK1 is the prototype of the PLK family with a well-defined role in cell cycle progression and cancer while PLK4 is important in centrosome dynamic during the cell cycle. PLK5 is kinase-defective due to a truncated kinase domain (Archambault and Glover, 2009; Andrysik et al., 2010; de Carcer et al.,2011a,b; Xu et al., 2012; Zitouni et al., 2014). PLK5 may function in cell cycle progression and the DNA damage response (Andrysik et al., 2010; de Carcer et al., 2011a). It appears to be restrictively expressed in the brain and involved in neuron differentiation, and progression of glioblastoma (Andrysik et al., 2010; de Carcer et al.,2011a,b; Xu et al., 2012; Zitouni et al., 2014). The functions of PLK2 and PLK3 are more diverse, with roles in stress response and cell cycle progression (Archambault and Glover, 2009; de Carcer et al., 2011b; Xu et al., 2012; Zitouni et al., 2014). All members of the PLK kinase family have been implicated in tumorigenesis as tumor promoters or suppressors (Eckerdt et al., 2005; Xu et al., 2012, 2017; Liu, 2015; Helmke et al., 2016).

PLK3 is involved in multiple events of cell cycle progression and checkpoint regulation in the DNA damage response following genotoxic stress (Xie et al., 2001, 2002; Bahassi El et al., 2002; Xu et al., 2012; Helmke et al., 2016). Later studies revealed functions of PLK3 in the cellular response to hypoxia (Wang et al., 2008, 2011; Xu et al., 2010, 2012; Li et al., 2017). Intriguingly, *Plk3* null mice have no overt abnormality (Yang et al., 2008), which is in contrast to the embryonically lethal phenotypes of *Plk1* or *Plk4* knockout in mice (Hudson et al., 2001; Lu et al., 2008). This suggests that other members of the PLK family may compensate for the loss of the function of PLK3 *in vivo*. However, *Plk3* null mice do show increased tumorigenesis in multiple organs at the advanced age (Yang et al., 2008). These observations indicate that regulation of stress responses, which may contribute to chronic conditions such as cancer, could be the primary function of Plk3 in mammals. Supporting its role in cancer, PLK3 expression tends to be lower in many human malignancies, including those in the lung, head and neck, colon, kidney, liver, stomach, breast, and rectum (Li et al., 1996; Xu et al., 2012; Helmke et al., 2016).

Murine embryonic fibroblasts (MEFs) from *Plk3* null mice exhibit a much higher level of HIF-1α in response to hypoxia or nickel, a hypoxia mimic, than wild-type MEFs (Yang et al., 2008; Xu et al., 2010). Ectopically expressed PLK3 suppresses nuclear accumulation of HIF-1α. Inhibition of hypoxia-induced HIF-1α nuclear translocation depends on the kinase activity of PLK3. Consequently, expression of VEGF-A, a major HIF-1 response protein, was also higher in *Plk3^–/–^* MEFs (Yang et al., 2008). Two evolutionarily conserved serine residuals of HIF-1α (i.e., Ser-576 and Ser-657) were identified as the PLK3 phosphorylation sites using *in vitro* kinase assay combined with mass spectrometry. Phosphorylation of these residuals destabilizes HIF-1α in a hydroxylation- and VHL-independent manner (Xu et al., 2010).

Recent work showed that hypoxia or nickel lowers the PLK3 protein level through UPS, with SIAH2 to be its ubiquitin E3 ligase (Li et al., 2017). PLK3 appears to interact with SIAH2 through two regions containing the potential consensus SIAH2 binding motif (House et al., 2003, 2006; Li et al., 2017). One of the motifs is located within the kinase domain whereas the other one resides slightly N-terminal of the PBD domain. The motif near PBD contributes more to the interaction with SIAH2 and PLK3 degradation (Li et al., 2017). PLK3, in turn, destabilizes SIAH2 in a kinase activity-dependent manner although the PLK3 phosphorylation site of SIAH2 remains to be identified (Li et al., 2017). PLK3 apparently promotes both auto-ubiquitination of SIAH2 and ubiquitination by another E3 ligase, as E3 ligase-deficient SIAH2 only partially prevents PLK3-mediated degradation (Li et al., 2017). This study discovered a mutual regulatory mechanism between PLK3 and SIAH2, which, along with the direct regulation of HIF-1α by PLK3 discovered before (Xu et al., 2010), may function to fine-tune HIF-1-mediated transcription and the cellular hypoxic response. In normoxia, PLK3 suppresses the hypoxic response by phosphorylating and destabilizing HIF-1α and SIAH2 (Xu et al., 2010). In hypoxia, the level and activity of SIAH2 increase via transcriptional and posttranslational mechanisms (Nakayama and Ronai, 2004; Nakayama et al., 2004, 2009; Qi et al., 2013). SIAH2 subsequently promotes degradation of PLK3 and PHDs, which helps maintain both HIF-1α and SIAH2 proteins at higher levels (Li et al., 2017).

### Regulation of SIAH2 by SRC

SRC is a protein tyrosine kinase with well-established roles in signaling pathways mediated by receptor tyrosine kinases, GPCRs, and integrins, leading to cell proliferation, cell cycle progression, and cell migration (Parsons and Parsons, 2004). It is one of the earliest and thoroughly documented proto-oncogenes (Irby and Yeatman, 2000). SRC is the prototype of the non-receptor tyrosine kinase family. Dysregulation of these kinases often leads to malignant transformation of cells (Parsons and Parsons, 2004).

SRC phosphorylates SIAH2 at tyrosine residues 86, 140, and 263 (Sarkar et al., 2012; Figure 1 and Table 1). Phosphorylation by SRC activates SIAH2, which is necessary and sufficient for SRC-mediated degradation of CCAAAT/enhancer binding protein delta (C/EBPδ), a protein with tumor suppressive function (Sarkar et al., 2012). Consistently, mutation of these residues prevents SIAH2-mediated degradation of C/EBPδ in MCF-10A mammary epithelial cells (Sarkar et al., 2012). Degradation of C/EBPδ mediated by SIAH2 leads to increased cell proliferation, migration, and invasion of breast cancer cells (Sarkar et al., 2012). Phosphorylation of SIAH2 by SRC apparently increases its activity toward C/EBPδ without affecting the expression level of SIAH2 in breast cancer cells. Consistently, inhibition of SRC using SKI-606, an inhibitor of the SRC family kinases, has no effect on the expression of SIAH2 in these cells. However, ectopic expression of an active SRC increases the protein level of SIAH2 in MCF-10A mammary epithelial cells (Sarkar et al., 2012). These observations suggest that breast cancer cells have lost their ability to regulate SIAH2 expression, which could be partly due to the much higher basal expression levels of SIAH2 in the breast cancer cells (Sarkar et al., 2012). Although SRC is known to be activated by hypoxia (Finn, 2008), the role of SRC in the SIAH2-HIF-1 axis has not been investigated. It is unclear whether the phosphorylation of SIAH2 by SRC affects its activity toward PHDs.

## Knowledge Gaps and Additional Consideration on the Regulation of the SIAH2-HIF-1 Axis by Protein Kinases

A recent report shows that phosphorylation of SIAH2 at Serine 6 and threonine 279 is correlated with the level of the serine/threonine kinase myotonic dystrophy kinase-related CDC42-binding kinase β (MRCKβ), suggesting SIAH2 may be a substrate of this kinase (Dixit et al., 2021). However, more evidence is need to confirm the direct interaction of the two proteins and direct phosphorylation of SIAH2 by MRCKβ. Although Plk3 is clearly a kinase that phosphorylates SIAH2, the phosphorylation sites have not been identified. Further study is clearly needed to answer these questions. Although SRC has been shown to phosphorylate SIAH2, the involvement of SRC in the SIAH2-HIF-1 axis is unclear at this time. As SRC is known to be activated by hypoxia (Finn, 2008), it is conceivable that SRC may contribute to the activation of SIAH2 in hypoxia. It is important to clarify this and to determine whether phosphorylation of SIAH2 by SRC affects its activity toward PHDs.

Several of the kinases that regulate SIAH2 are also subjected to regulation by SIAH2 through UPS. This is interesting but not surprising given that SIAH2 is an E3 ubiquitin ligase and that this type of feedback regulation makes biological sense. However, despite the effort of identifying the phosphorylation site on SIAH2 by the protein kinases, little attention has been focused on identifying the ubiquitination sites of the protein kinases by SIAH2. These sites should be equally important as the phosphorylation sites in understanding these pathways. In addition, although some work has been done, the interaction domains between the protein kinases and SIAH2 need to be thoroughly investigated. The importance of these aspects is highlighted by the findings that although SIAH2 also interacts with PHD2, it does not mediate its degradation. The mechanism underlying this observation could be determined by comparing the potential ubiquitination sites and/or SIAH2-interaction domains between PHD2 and PHD1/3. In addition, protein phosphorylation is a two-way process. When evaluating phosphorylation of SIAH2 by protein kinases, we must also consider the potential dephosphorylation process carried out by protein phosphatases. The study in this area is apparently lacking. As a result, no phosphatase has been reported to dephosphorylate SIAH2. Research in this area is clearly needed.

Studies in the past have mainly focused on the regulation of SIAH2 by protein kinases at the posttranslational level. Very little is known on whether and how these kinases regulation SIAH2 at the transcriptional level and how SIAH2 is induced transcriptionally under hypoxia. Although some evidence shows that SIAH2 can be transcriptionally induced in a PI3K/AKT-dependent fashion under hypoxia, the detailed mechanism remains to be determined. As PI3K pathway is known to be activated by ROS and hypoxic condition can trigger ROS production (Koundouros and Poulogiannis, 2018; Perillo et al., 2020), a likely mechanism is that PI3K/AKT pathway is activated by ROS in hypoxia, thereby promoting the transcription of SIAH2 by activating the downstream transcriptional events. Likewise, p38 and SRC can also be activated by ROS (Aikawa et al., 1997; Emerling et al., 2005). It is conceivable these kinases may also contribute to the transcriptional induction of SIAH2 under hypoxia. Other hypoxia-responsive events may also contribute to the activation of SIAH2 transcription. Exploring these mechanisms is clearly important for understanding the broader role of the SIAH2-HIF-1 signaling axis in the hypoxic response under physiological and pathological conditions.

## Regulation of the SIAH2-HIF-1 Axis by Protein Kinases and Cancer

The hypoxic response is deeply involved in tumorigenesis and tumor progression (Semenza, 1999, 2000; Harris, 2002). Elevated levels of HIF-1α are often found in tumors, which triggers neoangiogenesis that supplies nutrients and oxygen to the tumor mass by increasing the expression of angiogenic factors (Semenza, 1999, 2000; Bruick, 2003; Kaelin and Ratcliffe, 2008). The hypoxic response also orchestrates cellular changes that promote survival, sustain proliferation, and elicit metabolic reprogramming to adapt to the hypoxic tumor microenvironment (Semenza, 1999, 2000). The hypoxic response from tumor cells and stroma cells also reshapes the tumor microenvironment that promotes dormancy, stemness, invasion, and metastasis of cancer cells (Qiu et al., 2017). Given the importance of the hypoxic response in cancer, inhibition of the pathways that regulate this process has been increasingly recognized as an important strategy of cancer therapy (Semenza, 2003, 2012b).

SIAH2 is a ubiquitin E3 ligase with multiple cellular targets. Its functions in cancer biology are conceivably complex and context-dependent. Mounting evidence supports the role of SIAH2 as a tumor promoter in multiple types of cancer, including melanoma, prostate cancer, pancreatic cancer, breast cancer, gastric cancer, and lung cancer (Schmidt et al., 2007; Ahmed et al., 2008; Qi et al., 2008, 2013; Moller et al., 2009; Nakayama et al., 2009; Shah et al., 2009; Wong and Moller, 2013; Moreno et al., 2015; van Reesema et al., 2016; Kokate et al., 2018). For instance, SIAH2 serves as an oncoprotein with elevated expression in lung cancer (Qi et al., 2013; Wong and Moller, 2013; Moreno et al., 2015). The SIAH2 mRNA level in non-small cell lung cancer is elevated and positively associated with the grade and poor prognosis of the patients (Moreno et al., 2015). SIAH2 promotes degradation of Sprouty 2, a negative regulator of the Ras pathway, as well as the hypoxic response pathway to promote tumorigenesis (Nakayama et al., 2009; Wong and Moller, 2013). A recent study has linked SIAH2 to the cellular response to oxidative stress, remodeling of the tumor microenvironment, and tumor progression by targeting NRF1 for degradation in breast cancer (Ma et al., 2019). Another recent study has implicated SIAH2 in immune evasion of melanoma by promoting Treg proliferation via degradation of p27 (Scortegagna et al., 2020).

**FIGURE 2:**
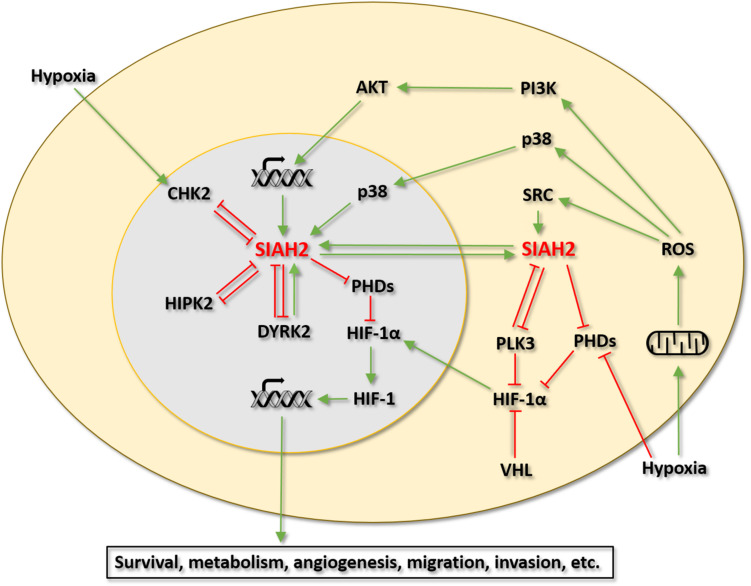
Regulation of the SIAH2-HIF-1 axis by protein kinases. Green arrows indicate activation. Red T bars indicate inhibition.

SIAH2 has emerged as an important regulator of the HIF-1 pathway and cellular response to hypoxia. Evidence collected thus far indicates that SIAH2 is directly regulated by at least 6 protein kinases. Among these kinases, p38, HIPK2, DYRK2, CHK2, and PLK3 have been linked to the regulation of HIF-1 signaling via SIAH2. Although not yet been examined, it would not be surprising that the regulation of SIAH2 by SRC also has significant implications in the HIF-1 pathway and the hypoxic response. Of interest, the four protein kinases that show negative regulation of SIAH2 (activity and/or stability), namely HIPK2, DYRK2, CHK2, and PLK3, are all considered to have tumor suppressive functions and with roles in the DNA damage response (Gwack et al., 2006; Sombroek and Hofmann, 2009; Li et al., 2017). All these kinases also have mutual regulatory relationships with SIAH2. These findings raise a notion that SIAH2 may participate in the tumor suppressive functions of these kinases by inhibiting the HIF-1 pathway and the hypoxic response in a feed forward fashion. For instance, HIPK2 may activate p53 by phosphorylating p53 at Serine 46 on the one hand and inhibit HIF-1 via SIAH2 on the other hand. In contrast, the two kinases that positively regulates SIAH2, namely p38 and SRC, both have well-established tumor promoting functions (Irby and Yeatman, 2000; Igea and Nebreda, 2015). Along this line of reasoning, SIAH2 may contribute to the tumor promoting functions of these kinases by activating the HIF-1 pathway and the hypoxic response. This notion would position SIAH2 at the crossroad of several important oncogenic pathways to coordinate the interaction between these pathways and the cellular hypoxic response (Figure 2).

## Potential of Targeting the SIAH2-HIF-1 Axis and Its Kinase Regulators for Cancer Therapy

Given the role of SIAH2 in the hypoxic response and cancer, this E3 ubiquitin ligase and its kinase regulators may be targeted for cancer therapy. The kinases that regulate SIAH2 are all considered druggable, with relatively well-established small molecule inhibitors (Table 2). Although inhibiting a ubiquitin E3 ligase such as SIAH2 is more challenging, some inhibitors of SIAH2 have, nonetheless, been identified (Shah et al., 2009; Stebbins et al., 2013; Feng et al., 2019; Table 2). The therapeutic values of the inhibitors targeting SIAH2 and its kinase regulators with respect to the SIAH2-HIF-1 signaling axis, when used alone or combined remain to be thoroughly evaluated. It is conceivable that inhibition of SIAH2 and/or the kinases that regulate SIAH2 may change the response of cancer cells to the hypoxic condition, thereby altering the trajectory of cancer progression. These changes could either produce therapeutic effects directly or open new therapeutic windows for other therapies.

**TABLE 2:** Representative inhibitors of SIAH2 and protein kinases that regulate SIAH2.

Target	Inhibitor	IC_50_	References
SIAH2	BI-107F7	20 μM	Shah et al., 2009
	BI-107F9	5–10 μM	Shah et al., 2009
	Menadione	5–10 μM	Feng et al., 2019
	Betulinic acid	0.1 μM	Stebbins et al., 2013
	Adapalene	0.1 μM	Stebbins et al., 2013
p38	SB203580	<1 μM	Young et al., 1997
	LY2228820	5.3–3.2 nM	Fisk et al., 2014; Cicenas et al., 2017
	SCIO-469	9–90 nM	Fisk et al., 2014; Cicenas et al., 2017
	BIRB-796	38–520 nM	Fisk et al., 2014; Cicenas et al., 2017
	PH-797804	26–102 nM	Fisk et al., 2014; Cicenas et al., 2017
	VX-745	10–220 nM	Fisk et al., 2014; Cicenas et al., 2017
HIPK2	TBID	0.33 μM	Yamada et al., 2009
DYRK2	Curcumin	5 nM	Nguyen et al., 2017; Jarhad et al., 2018
	BINDY	7.94 nM	Nguyen et al., 2017; Jarhad et al., 2018
	LDN192960	13 nM	Banerjee et al., 2019
CHK2	AZD7762	5 nM	Zabludoff et al., 2008
	NSC1095554	0.22–24 μM	Jobson et al., 2007; Jobson et al., 2009
	NSC744039	0.22–24 μM	Jobson et al., 2007; Jobson et al., 2009
	2-arylbenzimidazole	15 nM	Arienti et al., 2005
	VRX0466617	120 nM	Carlessi et al., 2007
	Isothiazole carboxamidine	0.09–0.46 μM	Larson et al., 2007
	CCT241533	3 nM	Anderson et al., 2011
Plk3	Poloxipan	3.2 μM	Kumar and Kim, 2015
SRC	Dasatinib	0.55 nM	Sen and Johnson, 2011; Levitzki, 2013
	Saracatinib	<4 nM	Sen and Johnson, 2011; Levitzki, 2013
	Bosutinib	<4 nM	Sen and Johnson, 2011; Levitzki, 2013

### SIAH2 Inhibitors

The role of SIAH2 in HIF-1 and Ras signaling positions SIAH2 as a potentially high-value target for cancer therapy. However, targeting an E3 ligase is difficult due to its lack of a well-defined catalytic domain. An inhibitor of SIAH2 would have to be able to interrupt the interaction between SIAH2 and its substrates or other components of the ligase complex in cases that SIAH2 works as an E3 ligase in complex with other proteins. Although continuing effort has identified a number of SIAH2 inhibitors (Table 2), the therapeutic values of these inhibitors in cancer therapy remain to be evaluated in depth.

Vitamin K3 (Menadione, MEN), a synthetic analog of vitamin K, is the first known small molecule inhibitor of SIAH2, identified by screening a library of 1840 compounds (Shah et al., 2009). Cellular work showed strong inhibition of SIAH2 self-ubiquitination/degradation and increase in the downstream targets of SIAH2, namely HIF-1α and Sprouty2, by MEN or its derivative (Shah et al., 2009). The inhibitory effect was not observed in SIAH2 knockout cells, confirming its specificity (Shah et al., 2009). MEN inhibits SIAH2 with an IC_50_ of 20 μM (Shah et al., 2009; Table 2). Notably, the inhibition was not affected by the free radical scavenger N-acetyl cysteine, suggesting that the effect of MEN is not caused by induction of free radicals, the known products of MEN treatment (Criddle et al., 2006; Shah et al., 2009). MEN has been shown to inhibit melanoma progression in the subcutaneous xenograft model and attenuate chemo resistance of chronic myeloid leukemia in the hypoxic microenvironment (Shah et al., 2009; Huang et al., 2018). However, when evaluating the therapeutic value of MEN, its ability to generate free radicals should not be overlooked. This effect could be a double-edged sword. On the one hand, it may potentiate the acute effect of MEN to kill cancer cells, particularly at high doses. On the other hand, it could facilitate tumor growth and metastasis at the low doses by regulating the stroma cells (e.g., cancer-associated fibroblasts) as well as cancer cells in the tumor microenvironment (Perillo et al., 2020).

Using a combination of Affinity Selection-Mass Spectrometry, a protein thermal shift-based assay, and an *in silico*-based screen, a number of small molecular inhibitors of SIAH2 have recently been identified (Feng et al., 2019). The top two hits that preferentially inhibit SIAH2 are a derivative of betulinic acid and adapalene (Feng et al., 2019). These compounds inhibit SIAH2 at the micromolar level of IC_50_ based on the cell viability assay using several melanoma and prostate cancer cell lines, with adapalene being a stronger inhibitor (Feng et al., 2019; Table 2).

Inhibition of SIAH2 using the polypeptide approach has also been explored. The rationale is to use a small peptide to interfere the binding between SIAH2 and its scaffold proteins or its substrates. Thus, overexpression of a polypeptide containing the first 130 amino acids of phyllopod, a *Drosophila* Siah scaffold protein, inhibits Siah-induced substrate degradation as well as breast cancer progression and melanoma metastasis (Qi et al., 2008; Moller et al., 2009). A shorter cell-penetrating polypeptide of 23 amino acids corresponding to amino acid 108-130 of phyllopod, which binds to the substrate binding domain of Siah, was able to inhibit the ubiquitin ligase activity of Siah in cultured cells (Moller et al., 2009). With this information, a recent study using a strategy based on rational structure-based design identified a number of peptides that covalently bind to SIAH2, thereby inhibiting the interaction between SIAH2 and its substrate without affecting its self-ubiquitination. The most potent one is BI-107F7 and its cell permeable derivative BI-107F9, which inhibit SIAH2 at an IC_50_ of 0.1 μM (Stebbins et al., 2013; Table 2). BI-107F9 was able to reduce the HIF-1α level in SW1 mouse melanoma cells maintained under hypoxia (Stebbins et al., 2013).

### Inhibitors of Protein Kinases That Regulates SIAH2

#### p38 Inhibitors

The involvement of p38 in a broad spectrum of cellular processes and multiple human pathological conditions, particularly inflammatory diseases and cancer, suggests that it may serve as an excellent therapeutic target (Wagner and Nebreda, 2009; Kyriakis and Avruch, 2012; Koul et al., 2013; Young, 2013; Fisk et al., 2014; Igea and Nebreda, 2015; Asih et al., 2020). Therefore, there has been significant interest in its inhibitors. Since the discovery of the first inhibitors of p38, namely SB203580 and SB202190, more than 20 years ago (Young et al., 1997), a few p38 inhibitors with improved selectivity and safety profiles have been developed and explored for the treatment of, among other human conditions, cancer (Fisk et al., 2014; Cicenas et al., 2017). These inhibitors are either type I (ATP competitive) or type II (allosteric inhibitors) with nanomolar IC_50_ levels (Cicenas et al., 2017). These inhibitors also inhibit different isoforms of p38 (i.e., p38α-δ) with different potency (Cicenas et al., 2017). Several clinical trials are underway for the treatment of various types of cancer using p38 inhibitors, including LY2228820, SCIO-469, BIRB-796, PH-797804, and VX-745 (Fisk et al., 2014; Cicenas et al., 2017; Table 2).

#### HIPK2 Inhibitors

HIPK2 has been increasingly recognized as a valid therapeutic target for cancer and renal fibrosis due to its role in the p53 pathway and TGF-β pathway. However, relatively limited progress has been made on its inhibitors to date. The p38 inhibitor SB203580 was initially regarded as also an inhibiter of HIPK2 due to the similarity of these two kinases (Di Stefano et al., 2004; Roscic et al., 2006; Yamada et al., 2009). However, a later study comparing its effect on 71 protein kinases showed that this compound (at 1 μM) has limited effect on the HIPK2 kinase activity while strongly inhibits 6 other protein kinases (Cozza et al., 2014). This study puts the selectivity of SB203580 toward HIPK2 in question. Using a rational design approach, this group developed an inhibitor of HIPK2 based on inhibitors of CK2 (Yamada et al., 2009). This compound, named TBID, exhibited strong selectivity toward HIPK2, with an IC_50_ of 0.33 μM (Yamada et al., 2009; Table 2).

#### DYRK Inhibitors

The search for inhibitors of the DYRK family kinases has been focused on DYRK1A due to its more established role in cancer and neurological conditions (Aranda et al., 2011; Soppa and Becker, 2015; Boni et al., 2020). A number of DYRK1A inhibitors have been identified with various selectivity (Nguyen et al., 2017; Jarhad et al., 2018). These inhibitors belong to several classes of compounds: pyrimidine derivatives, quinolones and quinazolines, indole derivatives, benzothiazole derivatives, imidazoles, harmine derivatives, and imidazolones (Nguyen et al., 2017; Jarhad et al., 2018). The similarity between DYRK1A and DYRK2 allows some of these inhibitors to also inhibit DYRK2, albeit with different potency compared to DYRK1A (Nguyen et al., 2017; Jarhad et al., 2018; Table 2). For instance, harmine inhibits all members of the DYRK family, although with higher selectivity toward DYRK1A. Curcumin, an active ingredient of *Curcuma longa*, has been shown to be more selective toward DYRK2 (with IC_50_ of 5 nM) over other members of the DYRK family. BINDY, a benzothiazole derivative, more potently inhibits DYRK2 (IC_50_ = 7.94 nM) than DYRK1A (IC_50_ = 25.1 nM) (Nguyen et al., 2017; Jarhad et al., 2018). A recent study identified another selective DYRK2 inhibitor named LDN192960 with an IC_50_ of 13 nM (Banerjee et al., 2019). This compound has a significant advantage over curcumin, which is hydrophobic, in that it is hydrophilic. This feature allows more cell permeability and makes it a more attractive drug candidate (Banerjee et al., 2019). This compound is able to inhibit proliferation and invasion, induce cell death of triple negative breast cancer cells, and blunt tumor progression of these cells in xenograft model (Banerjee et al., 2019).

#### CHK2 Inhibitors

Significant effort has been made on identifying CHK2 inhibitors due to the strong involvement of CHK2 in cancer and the potential for combined therapy with other chemotherapeutic drugs. AZD7762 (thiophene carboxamide urea), discovered as a CHK1 inhibitor, inhibits CHK2 and CHK1 with approximately equal potency (IC_50_ = 5 nM) (Zabludoff et al., 2008). More selective inhibitors of CHK2 include NSC1095554 and its derivative NSC744039 (PV1019) (IC_50_ = 0.22 – 24 μM) (Jobson et al., 2007, 2009), 2-arylbenzimidazole (IC_50_ = 15 nM) (Arienti et al., 2005), VRX0466617 (IC_50_ = 120 nM) (Carlessi et al., 2007), a series of isothiazole carboxamidine compounds (IC_50_ = 0.087 – 0.46 μM) (Larson et al., 2007), CCT241533 (IC_50_ = 3 nM) (Anderson et al., 2011), pyrazole-benzimidazole conjugates (IC_50_ = 5.5 nM – 67.07 nM) (Galal et al., 2017, 2018a), and pyrimidine-benzimidazole conjugates (IC_50_ = 5.56 nM – 46.20 nM) (Galal et al., 2018b; Table 2). Some of these compounds (e.g., CCT241533 and NSC744039) have been shown to potentiate the effect of chemotherapeutic drugs in cultured cancer cells (Jobson et al., 2009; Anderson et al., 2011).

#### PLK3 Inhibitors

Searching for inhibitors of the PLK family kinases has been focused on PLK1 due to its well-established role in cancer as a tumor promotor (Strebhardt, 2010; Murugan et al., 2011; Liu, 2015). Several PLK1 inhibitors, both ATP competitive and non-ATP competitive, are in preclinical or clinical trials for cancer therapy, including Rigosertib (ON 01910), Volasertib (BI6727), GSK461364, GW843682, PPG, Onvansertib (NMS-P937), BI2536, MLN0905, Ro3280, Cyclapolin1, and SBE 13 (Murugan et al., 2011; Kumar and Kim, 2015; Liu, 2015). These inhibitors typically have nanomolar IC_50_ levels toward PLK1 (Murugan et al., 2011; Kumar and Kim, 2015; Liu, 2015). Majority of these inhibitors also inhibits PLK3, albeit with at least four times higher IC_50_ levels. Among them, GW843682 has the closest IC_50_ level toward PLK3 compared to that of PLK1 (9.1 nM for PLK3 vs. 2.2 nM for PLK1) (Murugan et al., 2011; Liu, 2015). Attempts to enhance the specificity toward PLKs over other kinases or PLK1 over other PLKs have been made to inhibit its PBD using small molecules, phosphopeptides, or using siRNA (Kumar and Kim, 2015; Hymel et al., 2018; Alverez et al., 2020). For instance, small chemicals Poloxin and poloxipan inhibit the polo box of PLKs with the IC_50_ ranging from 1.17 μM to 53.9 μM toward PLK 1/2/3. Poloxipan has about equal potency toward PLK1 and PLK3 (IC_50_ of 3.0 μM for PLK1 and 3.2 μM for PLK3) (Table 2) whereas Poloxin is more selective toward PLK1 (IC_50_ of 4.8 μM for PLK1 and 53.9 μM for PLK3) (Kumar and Kim, 2015). TKM-080301, an siRNA-based PLK1 inhibitor specifically targets PLK1 and inhibits xenograft tumors (Kumar and Kim, 2015). An inhibitor that preferentially inhibits PLK3 is clearly needed in order to selectively target PLK3.

#### SRC Inhibitors

A few tyrosine kinase inhibitors (TKIs) with activity toward SRC have been identified to date. SRC inhibitors tend to also inhibit other members of the SRC family tyrosine kinases, as well as receptor tyrosine kinases (Sen and Johnson, 2011; Levitzki, 2013). Some of these are either in clinical trials or have been marketed to treat various types of cancer. For instance, Dasatinib (BMS-354825), a small molecular inhibitor of multiple tyrosine kinases with an IC_50_ of 0.55 nM toward SRC and the similar IC_50_ for other tyrosine kinases of the SRC family, has been shown to inhibit the progression of chronic myelogenous leukemia (CML) and other cancer types (Sen and Johnson, 2011; Levitzki, 2013). Other prominent TKIs include Saracatinib (AZD0530) with an IC_50_ of < 4 nM for SRC family tyrosine kinases and Bosutinib (SKI-606) with a similar IC_50_ toward the SRC family tyrosine kinases (Sen and Johnson, 2011; Levitzki, 2013; Table 2). These TKIs exhibit significant therapeutic effects on solid tumors (e.g., breast cancer) (Sen and Johnson, 2011; Levitzki, 2013).

## Conclusions and Perspectives

The list of protein kinases that regulate SIAH2 will likely become longer over time. The regulation of the HIF-1 pathway and the cellular hypoxic response by SIAH2 highlights the potential importance of this protein in converging inputs from multiple kinases to the HIF-1 pathway. This feature of the SIAH2-HIF-1 signaling axis combined with the fact that SIAH2 is a potentially druggable protein present some appealing therapeutic opportunities for cancer treatment. The therapeutic value of this signaling axis could be more attractive for combined therapy targeting the kinases that regulate SIAH2. In addition, the expression level of SIAH2 may have an important prognostic value in certain cancer types. However, in order to target the SIAH2-HIF-1 axis for cancer therapy, we must fully appreciate the complexity of the pathway and its downstream biological effects. The protein kinases that regulate SIAH2 all have multiple targets in addition to SIAH2. SIAH2, as an E3 ubiquitin ligase, regulates the degradation of many different cellular targets too. There are also mutual regulative mechanisms between SIAH2 and some of the protein kinases that regulate SIAH2 (Figure 2). A positive feedback relationship also exists between SIAH2 and the HIF-1 pathway. These characteristics present both challenges and opportunities in the understanding and targeting of this important signaling axis. Further studies in these areas should be a fruitful endeavor.

## Author Contributions

DX drafted the manuscript. CL participated in writing and editing the manuscript. Both authors contributed to the article and approved the submitted version.

## Conflict of Interest

The authors declare that the research was conducted in the absence of any commercial or financial relationships that could be construed as a potential conflict of interest.
